# Enhanced bone regeneration by low-intensity pulsed ultrasound and lipid microbubbles on PLGA/TCP 3D-printed scaffolds

**DOI:** 10.1186/s12896-023-00783-9

**Published:** 2023-06-06

**Authors:** Lin Jin, Jiali Shan, Yanhong Hao, Yingchun Wang, Liping Liu

**Affiliations:** 1 Department of Ultrasound, Jiading District Central Hospital Affiliated Shanghai University of Medicine & Health Sciences, Shanghai, 201800 China; 2 Department of Ultrasound, Guanghua Hospital Affiliated to Shanghai University of Traditional Chinese Medicine, Shanghai, 200052 China; 3 Department of Ultrasound, First Hospital of Shanxi Medical University, No. 85, Jiefang South Road, Taiyuan, 030001 Shanxi China

**Keywords:** Bone repair, Low-intensity pulsed ultrasound, Microbubble, 3D printing scaffold, Scaffold

## Abstract

**Background:**

To investigate the effect of low-intensity pulsed ultrasound (LIPUS) combined with lipid microbubbles on the proliferation and bone regeneration of bone marrow mesenchymal stem cells (BMSCs) in poly (lactic-glycolic acid copolymer) (PLGA)/α-tricalcium phosphate (TCP) 3D-printed scaffolds.

**Methods:**

BMSCs were irradiated with different LIPUS parameters and microbubble concentrations, and the best acoustic excitation parameters were selected. The expression of type I collagen and the activity of alkaline phosphatase were detected. Alizarin red staining was used to evaluate the calcium salt production during osteogenic differentiation.

**Results:**

BMSCs proliferation was the most significant under the condition of 0.5% (v/v) lipid microbubble concentration, 2.0 MHz frequency, 0.3 W/cm^2^ sound intensity and 20% duty cycle. After 14 days, the type I collagen expression and alkaline phosphatase activity in the scaffold increased significantly compared to those in the control group, and alizarin red staining showed more calcium salt production during osteogenic differentiation. After 21 days, scanning electron microscopy experiments showed that osteogenesis was obvious in the PLGA/TCP scaffolds.

**Conclusion:**

LIPUS combined with lipid microbubbles on PLGA/TCP scaffolds can promote BMSCs growth and bone differentiation, which is expected to provide a new and effective method for the treatment of bone regeneration in tissue engineering.

## Introduction

A variety of trauma, tumours, infections, and congenital bone diseases caused by large bone defects, which affect the quality of life and physical and mental health of patients, have been a common clinical problem [1]. It is estimated that more than 2 million bone grafts are performed worldwide each year to provide a solution for cases in which the natural repair of bone is hampered [2]. Tissue engineering has provided hope for bone repair and is an effective way to repair bone defects, which has been a concern of clinical and basic researchers [3]. However, it is difficult for traditional scaffold materials to have a good biocompatibility, biodegradability and porous three-dimensional structure and to have the characteristics of bone conduction, bone induction and osteogenesis [4, 5]. The development of biomaterials, stem cells and bone tissue engineering technology provide new hope for bone regeneration. 3D-printing technology can be designed for patients by computers, can adjust shapes and internal 3D structures, and can prepare personalized scaffolds with biocompatibility, which, in comparison to previous methods, can better solve the problem of personalized and accurate repair [6].

Low-intensity pulsed ultrasound (LIPUS) is a mechanical stimulation composed of constant periodic amplitude waves with intensity or sonic intensity (SI) ranging from 5 to 100 mW/cm^2^ [7]. Preclinical trials have demonstrated the potential of LIPUS in the field of bone tissue repair and regeneration. LIPUS not only acts on osteoblasts [8], osteoclasts [9] and mesenchymal stem cells [10] to exert osteogenic effects, but also has a positive effect on bone healing and regeneration through its effects on blood vessels and nerves [11, 12]. Ultrasonic lipid microbubbles (MBs) can burst to form acoustic cavitation under the action of ultrasound, and the energy released can start or promote the sonochemical reaction [13].

Therefore, we hypothesized that LIPUS combined with MBs could promote the proliferation and bone regenerative repair of bone marrow mesenchymal stem cells (BMSCs) in 3D-printed scaffolds. Furthermore, we focused on the influence of LIPUS combined with MBs in different irradiation intensity to promote bone repair, and finally got optimal acoustic parameters.

## Materials and methods

### Preparation of 3D-printed scaffolds

α- tricalcium phosphate (TCP) was synthesized by a mixture of CaCO_3_ and CaHPO_4_ in a certain ratio in a high temperature calcination reaction. A mixture of gelatin (10% (w/v)), glycerol (10% (v/v)), and glutaraldehyde (1% (v/v)) was applied as dispersant and binder. The mixture and PLGA (Mw = 200,000, Jinan Daigang Biomaterial Co., Ltd, China) were used as the two kinds of ink for 3D printing. After 3D printing, α-TCP was transformed into calcium-deficient hydroxyapatite (CDHA) through a hydration reaction. The 3D printing was performed in a double print-head mode with a 3D-bioplotter. Finally, the two components of PLGA/TCP were well combined to prepare a bilayer scaffold [14]. The inner diameter of the discharge needle was 0.5 mm, the printing pressure was 0.2 MPa. Then, the solution was naturally cured at room temperature for 24 h, and the scaffolds’ morphology were assessed by scanning electron microscopy (SEM, TESCAN Mira3).

### Culture of rat BMSCs

SD rats aged 3–4 weeks were obtained from the experimental animal center of Shanghai Rat&Mouse Biotech Co.,Ltd (Shanghai, China) and were euthanized by intraperitoneal injection of 200 mg/kg sodium pentobarbital and immersed in 75% alcohol for disinfection. The limbs were collected and peeled and then immersed in alcohol for approximately 3 min. The bones and soft tissue were cleaned by PBS 3 times. The bone marrow was washed with culture medium 2–3 times with a 5 ml syringe. A total of 250 g of the washing solution was collected and centrifuged for 10 min. The cells were cultured in complete culture medium (DMEM/F12, Thermo) and cultured in a cell culture dish for 24 h. The media were replaced every other day. Cells were incubated under standard cell culture conditions (37 °C, 5% CO_2_ and 95% relative humidity). The cells were divided and proliferated to 80 − 90% confluence and then passaged. Third-generation BMSCs were used in the experiment.

### MBs preparation

**Fig. 1:**
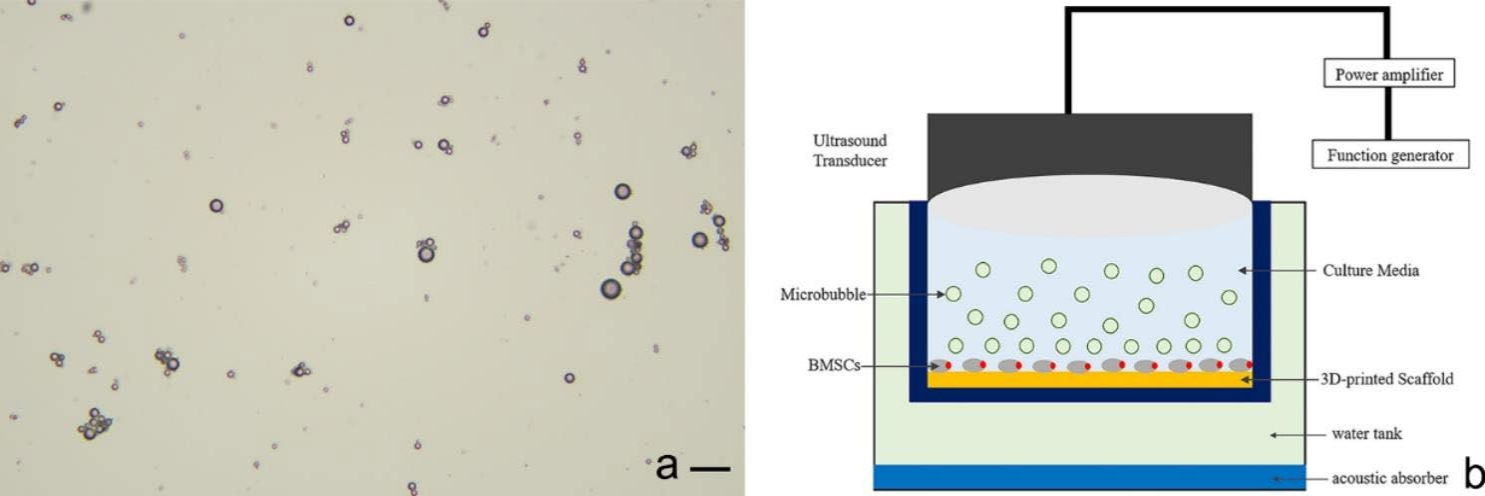
(**a**) Size distribution of lipid microbubbles (x400); (**b**) Irradiation method of LIPUS combined with lipid microbubbles on scaffolds. scale bar shows 25 μm

MBs (SonoVue™, Bracco, Italy) were used. MBs were formulated with distearyl phosphatidylcholine, sodium dipalmitoylphosphoglycerate, polyethylene glycol 4000 and palmitic acid, with a total lipid concentration of approximately 2.0 × 10^8^ particles/mL and an average microbubbles diameter of 2.5 μm. The morphology of MBs was observed under microscope (Olympus, Japan) (Fig. 1a). Then the MBs were diluted to different concentrations (0, 0.5, 1, 2, 4 and 5% v/v). MBs and BMSCs were cocultured to detect the microbubble cytotoxicity. Cells were incubated under standard cell culture conditions (37 °C, 5% CO_2_ and 95% relative humidity). The MBs were replaced every day. After 24 and 72 h, cell proliferation was detected by a CCK8 Kit (UBI, Yobio).

### LIPUS excitation

The low-frequency pulse ultrasonic therapeutic instrument was made by Shanghai Acoustics Laboratory, Chinese Academy of Sciences. The outside diameter of ultrasound transducer was 35 mm. The unfocused transducer was calibrated using a needle hydrophone (NH4000, PA, England) in a water tank filled with degassed deionized water. All LIPUS stimulations were carried out at a constant pulse repetition period of 10ms, the duty cycle (20%, continuous mode) and the excitation SI (0.1w/cm^2^ and 0.3 w/cm^2^) to determine the optimum acoustic setting. The corresponding area effective radiated sound power at those intensities were 1.10 w and 3.02 w. The average effective SI were 0.114 w/cm^2^ and 0.314 w/cm^2^. In this study, the transducer was sterilized with 75% alcohol, via ultraviolet (UV) exposure and then rinsed with PBS three times before the experiments.

### Irradiation of BMSCs by LIPUS combined with MBs

The optimal concentration of LBs was added to PBS solution, and a LIPUS therapeutic instrument was used to connect the ultrasonic probe for LIPUS irradiation. After 24 and 72 h of culture, the cell number was detected via a CCK-8 assay to screen the best irradiation parameters of LIPUS.

Samples were treated with varying LIPUS parameters for 3 min once. The group were divided as follows: Group A, control group, only MBs, no LIPUS irradiation; Group B, LIPUS groups, only LIPUS irradiation, no MBs; Group C, LIPUS + MBs group. Group B was further divided into the B1 group, low intensity group (frequency 1.0 MHz, SI 0.1w/cm^2^, 20% duty cycle); B2 group, medium intensity group (frequency 1.0 MHz, SI 0.3w/cm^2^, 20% duty cycle); B3 group, high intensity group (frequency 2.0 MHz, SI 0.3w/cm^2^, 20% duty cycle). Group C was further divided into the C1 group, low intensity group (frequency 1.0 MHz, SI 0.1w/cm^2^, 20% duty cycle); C2 group, medium intensity group (frequency 1.0 MHz, SI 0.3w/cm^2^, 20% duty cycle); C3 group, and high intensity group (frequency 2.0 MHz, SI 0.3w/cm^2^, 20% duty cycle).

### Irradiation of scaffolds by LIPUS combined with MBs

The scaffolds were sterilized via UV exposure, immersed in 75% alcohol for 2 h and then rinsed with PBS three times. Subsequently, the sterilized samples were pre-soaked in culture media for 24 h before cell seeding. The LIPUS transducer head was placed vertically on the top of the cell culture medium and touched the surface of the culture medium. The distances were maintained throughout all experiments. The optimal LIPUS parameters were used to stimulate BMSCs proliferation under scaffolds and MBs suspensions. To prevent the possibility of indirect energy transfer from LIPUS to the neighbouring wells, scaffolds were distributed in every other well of a 6-well plate (one empty well in between) with 2 × 10^5^ cells per well. Media were replaced every other day, and the irradiation method is shown in Fig. 1B. Consequently, the samples were divided into two groups: Group A (control group, no MBs, no LIPUS), Group B (LIPUS + MBs).

### Alkaline phosphatase (ALP) activity assay and quantitative polymerase chain reaction (qPCR)

To confirm the osteogenic potential of the LIPUS treatment, the expression of osteogenic markers collagen-I, ALP was studied fourteen days after induction of osteogenic differentiation. ALP activity was measured by a visible light colorimetry standard assay kit following the manufacturer’s instructions (Jiangcheng, Nanjing, China), and collagen-I was detected using qPCR. The total RNA was extracted from the cells by Trizol reagent (Invitrogen, China). For cDNA synthesis, preparation of Master Mix for reverse transcription reaction. The qPCR assays were performed using SYBR Green (ABI, China) on a fluorescence quantitative PCR instrument (Applied Biosystems, US).

### Alizarin red staining

To measuring the formation of calcium salt deposition during osteogenic differentiation, the alizarin red staining was used. The cells were fixed and stained for 20 min with Alizarin Red (Solarbio, China). The mineralized nodules of cells in various treatments were observed and images were captured using a microscope (Olympus, Japan). Image-Pro Plus version 6.0 software (Media Cybernetics, Inc., Rockville, MD, USA) was used to assess the area and density of the dyed region, and the integrated optical density value. The IOD from five randomly selected fields were assessed in a blinded manner and subjected to statistical analysis.

### Statistical analysis

All statistical analyses were performed with GraphPad Prism 9 software (GraphPad Software Inc., San Diego, CA, USA). The measurement data are expressed as mean ± standard deviation (SD), and t test and one-way ANOVA were used for comparing the measurement data between two groups and among multiple groups, respectively. *P* < 0.05 indicated that the difference was statistically significant.

## Results

### Characterization of 3D-printed scaffolds

**Fig. 2:**
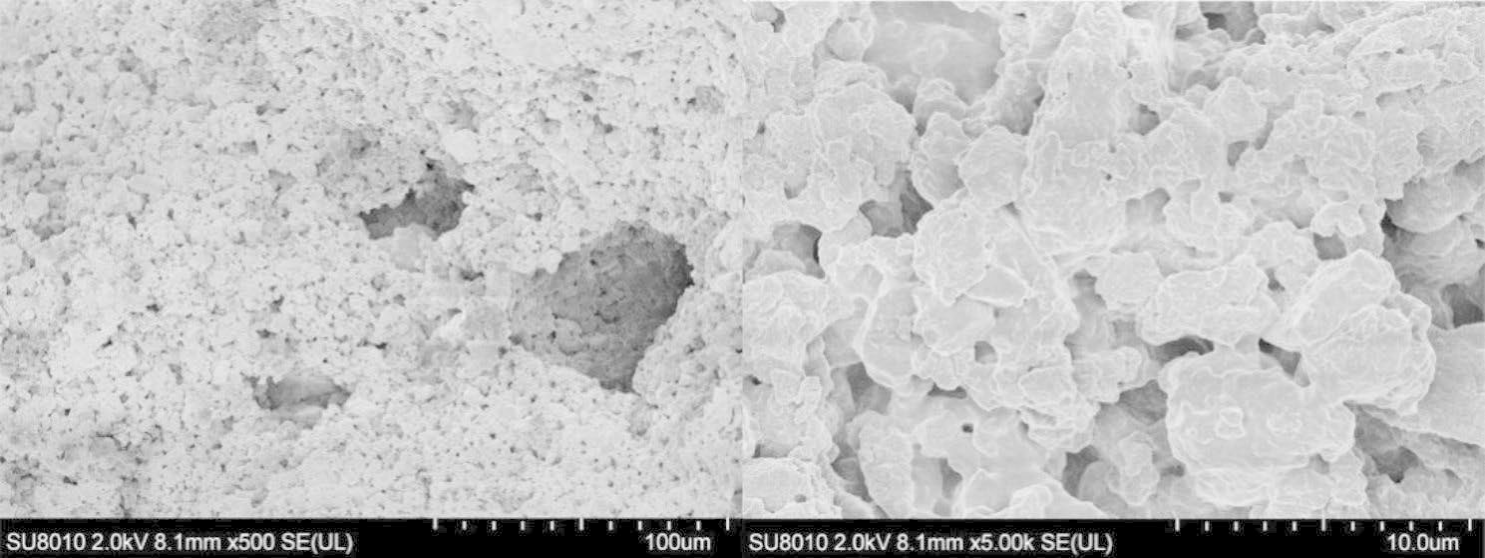
SEM images of the structure of CDHA/PLGA 3D-printing scaffold. The multi-layer pore structure of the scaffold and the closely arranged CDHA crystals were observed by SEM.

Typical SEM images of 3D-printed scaffolds are presented in Fig. 2. Pores can be seen on the material surface, the pores in the material are connected with each other, and a large number of scattered high-density particles can be seen on the material surface and in the pores.

### Morphological observation of BMSCs

After 12 h, the cells began to adhere to the well, and the cells were round or oval. After 48 h, most of the cells adhered to the well, but their morphologies were different. On the third day, the number of proliferating cells increased, and scattered adherent fibroblast-like cells appeared. On the Fifth to seventh day, the number of cells increased significantly, and the cells grew in a monolayer. The morphology of the cells tended to be uniform and were spindle. The passaged cells completely adhered to the well within 24 h. The third-generation cells were spindle or fusiform with uniform distribution and size.

### Optimal concentrations of MBs

**Fig. 3:**
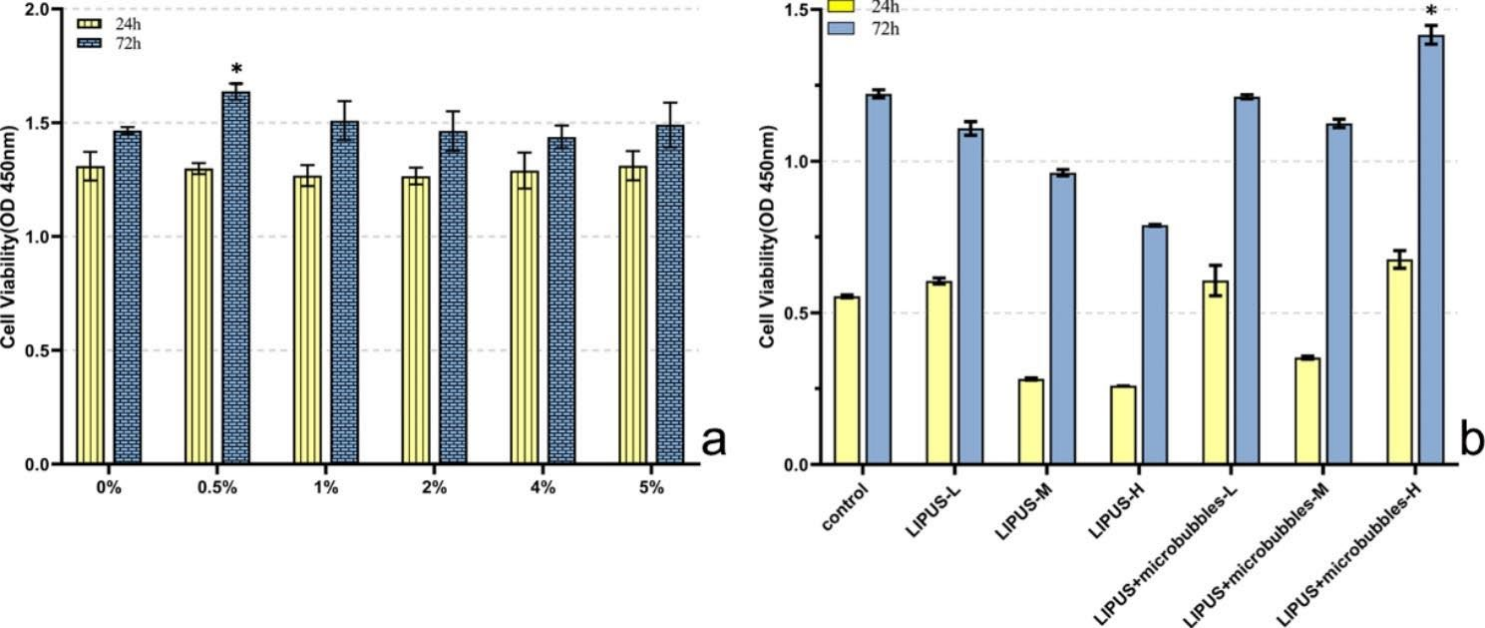
(**a**) 24 and 72 h cell viability of BMSCs incubated with microbubbles. It showed cells grew more with 0.5% microbubbles. Values significantly different from other groups are indicated by *for P < 0.05 (n = 3). (**b**) BMSCs proliferation after 24 h and72 hrs with different intensity LIPUS and LIPUS + MB. It showed LIPUS + MB (20% duty cycle, 2.0 MHz with 0.3w/cm^2^ LIPUS) leads to highest increase. Values significantly different from other groups are indicated by *for P < 0.05

According to the OD value measured at 450 nm, the results showed increased cell proliferation after both 24- and 72-hour time periods. The growth trend of the five groups of cells was basically the same, and the cell proliferation was gentle at 24 h. There was no significant difference in the number of cells in each group (*P* > 0.05). At 72 h, the cell proliferation rate of each group was accelerated, and the cell proliferation activity of the 0.5% (v/v) group was higher than that of the other groups (*P* < 0.05), as shown in Fig. 3a. The results indicated that MBs do not cause short-term or long-term cytotoxicity to BMSCs at the concentrations studied here. Therefore, the subsequent experiments were performed with 0.5% (v/v) MBs.

### Effects of LIPUS parameters on BMSCs proliferation

A 0.5% (v/v) MBs suspension was added to the cell culture medium to detect the proliferation of BMSCs 24 and 72 h after LIPUS stimulation at different intensities for 3 min. The results showed that LIPUS (frequency 2.0 MHz, SI 0.3 W/cm^2^, 20% duty cycle) in the presence of 0.5% (v/v) MBs suspension significantly increased cell proliferation after 72 h (*P* < 0.05). BMSCs proliferation was enhanced up to 15.77% compared to that of the control group after 72 h of culture. The results are presented in Fig. 3b. As a result, 20% duty cycle, 2.0 MHz with 0.3w/cm^2^ LIPUS excitation was used for all experiments.

### Effect of LIPUS and MBs on BMSCs differentiation in 3D-printed scaffolds

**Fig. 4:**
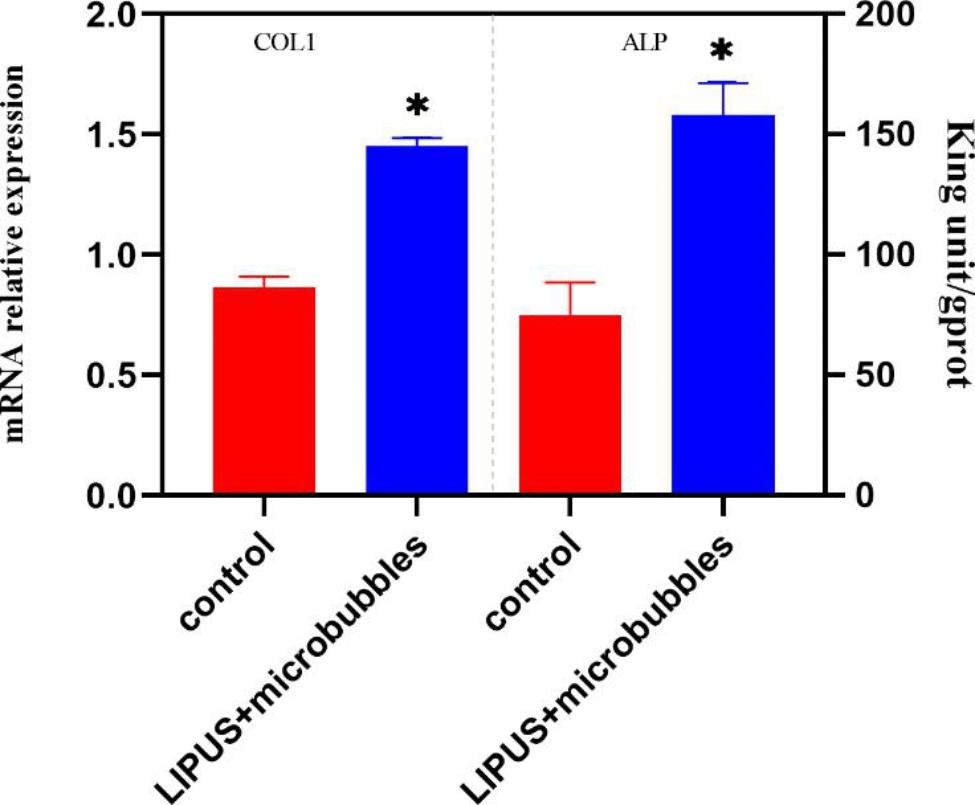
The expression of ALP (by light coliometry) and Type I collagen (by qPCR test) after 14d. The leves was normalized to the reference gene Actin (n = 3)

**Fig. 5:**
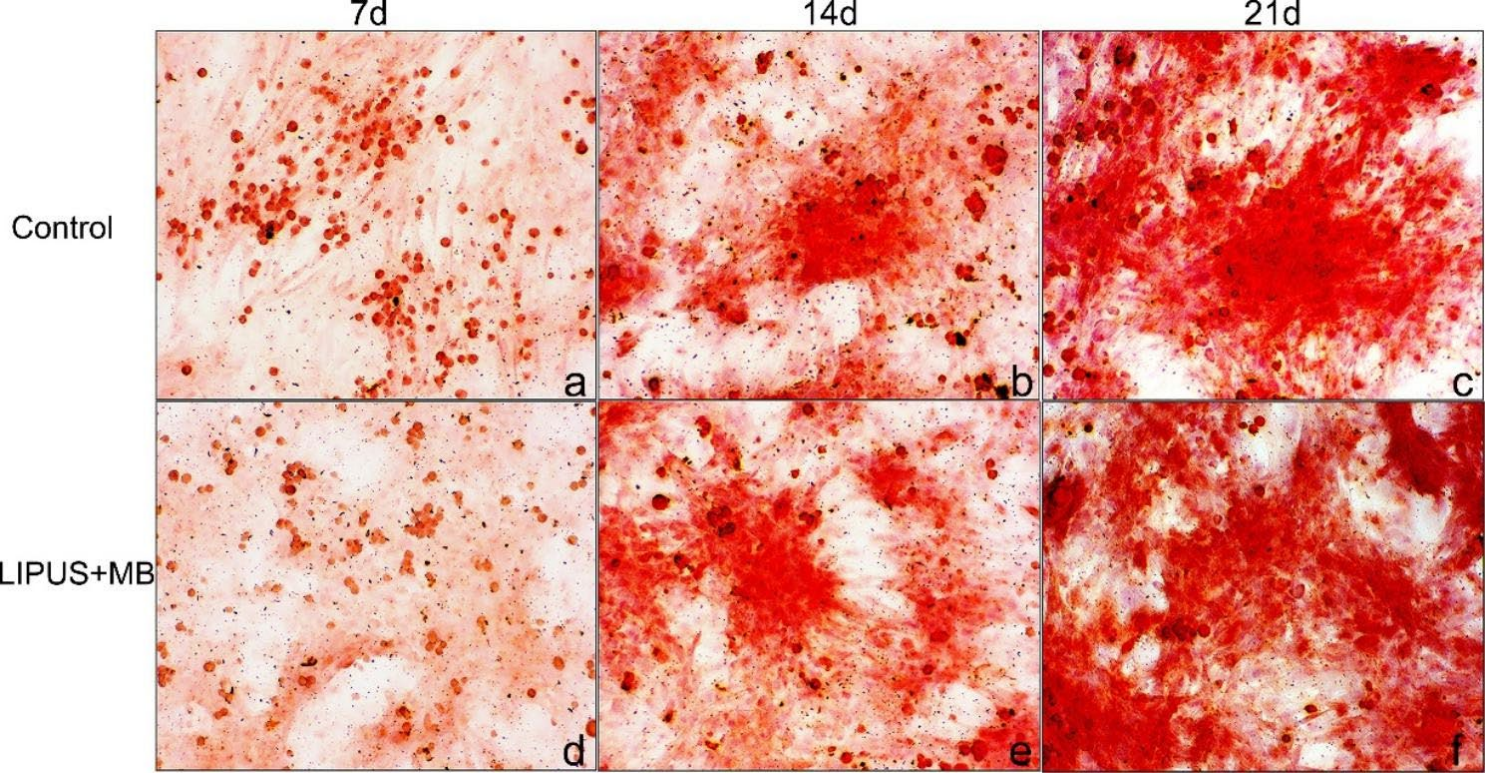
Alizarin red staining assay on 7, 14 and 21 days. (**a**) alizarin red staining of control on 7 days (magnification, x100); (**b**) alizarin red staining of control on 14 days (magnification, x100); (**c**) alizarin red staining of control on 21 days (magnification, x100); (**d**) alizarin red staining of LIPUS + MB group on 7 days (magnification, x100); (e) alizarin red staining of LIPUS + MB group on 14 days (magnification, x100); (**d**) alizarin red staining of LIPUS + MB group on 21 days (magnification, x100). The positive expression of Alizarin red staining in LIPUS + MB group was higher than that in the control group on 14 and 21 day

The osteogenic marker of ALP activity was increased by approximately 50% compared with controls (*P* < 0.05, Fig. 4). Similarly, the protein expression of collagen-I in microbubbles combined with LIPUS group was also increased. Consistently, the osteoblastic mineralization in the cells, as visualized by alizarin red staining, was significantly increased in LIPUS + MB group (Fig. 5). On the 7 days, the mean density of control group was 0.108 ± 0.004, and the LIPUS + MB group was 0.098 ± 0.002, while on the 21 days, the mean density of control group was 0.302 ± 0.003, and the LIPUS + MB group was 0.347 ± 0.011 (P<0.05).

**Fig. 6:**
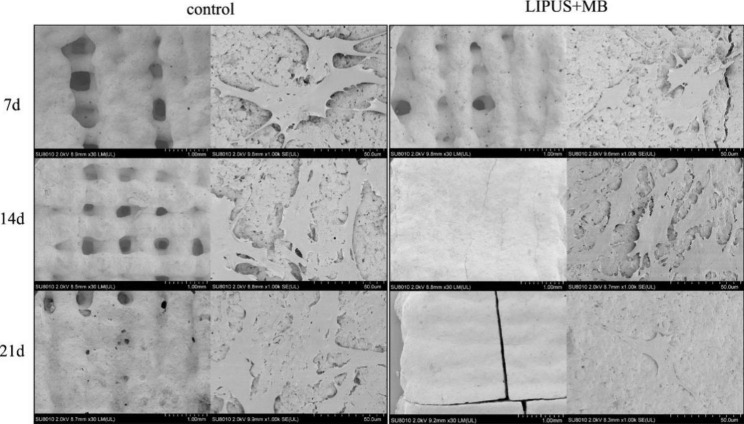
SEW images of scaffolds and BMSCs cultered 7,14 and 21 days. The pores of the scaffolds were covered, and osteogenesis was obvious in the LIPUS + MB group than in the control group. The numbers of cells in the LIPUS + MB group were higher than in the control group

To analyse the adhesion and proliferation of BMSCs on the scaffold, scanning electron microscopy was used to observed the scaffold (Fig. 6). The numbers of cells in the LIPUS + MB group were higher than those in the control group after 7 and 14 days of culture, and osteogenesis was obvious at 21 days. After 7 days of culture, the cells adhered well on the surface of the scaffold, the morphology was clear, and the cells grew intensively in some areas to form some cell clusters. After 14 days of culture, more cells proliferated, connected with each other, attached to the surface of the material and penetrated the pores of the material. When cultured for 21 days, the pores of the material were covered, and osteogenesis was obvious.

## Discussion

This study investigated the possibility of using LIPUS combined with lipid microbubble irradiation of BMSCs on PLGA/TCP 3D-printed scaffolds to promote bone regeneration. We found that LIPUS (frequency 2.0 MHz, sound intensity 0.3 W/cm^2^, 20% duty cycle) combined with microbubble (0.5% (v/v)) irradiation promoted more BMSCs growth and bone tissue regeneration.

Calcium phosphate biomaterials are considered to be a promising biomaterial for bone regeneration because of their inherent biocompatibility, biodegradation, and suitable elastic modulus close to bone [15]. Personalized 3D-printing scaffolds with improved bone defect repair can be made by using 3D-printing technology [16]. Many attempts have been made by scholars to functional modification and loading bioactive substances on calcium phosphate scaffolds for 3D-printing technology. However, the scaffolds suffered low mechanical property which limited their clinical application in bone regeneration [17, 18].

PLGA is a kind of degradable polyester with sufficient mechanical strength and good biocompatibility, which can be completely degraded in vivo. The PLGA/TCP 3D-printing scaffold has good biocompatibility and bone conductivity, is suitable for osteoblast adhesion and can promote the adhesion and proliferation of osteoblasts on the surface [19]. However, if cells are efficiently and evenly planted on such 3D-printing scaffolds with special structures, obtaining a high concentration and effective cell quantity is the key to tissue engineering [20].

Bone cells have a good response to mechanical stimulation caused by ultrasound, while the rate and extent of bone healing can theoretically be increased by incorporating physical or biochemical cues into the injury site [21]. LIPUS can promote bone effects by promoting cell proliferation and acting on osteoblasts and mesenchymal stem cells to repair bone defects [10, 22]. The biological effect of LIPUS is closely related to its time of action, intensity, frequency and other parameters. Therefore, determining the optimal ultrasonic parameters is very important when studying LIPUS combined with scaffolds for bone defect repair. Lipid microbubbles can be used to enhance ultrasound imaging and drug delivery [23]. Research showed that ultrasound can rupture the cell membrane and form transient pores on the cell membrane, which is conducive to gene entry into the cell, improves the efficiency of gene transmission, and does not cause cell damage [24, 25]. Indeed, lipid microbubbles and ultrasound have been utilized for the repair of bone tissue engineering [26, 27]. However, it is important to investigate effects of each individual component of the mechanical cues and environment, such as LIPUS, microbubbles, scaffold structure. In this study, BMSCs grew well under 0.5% (V/V) lipid microbubbles, indicating that lipid microbubbles had little toxic effect on cells. Moreover, LIPUS combined with lipid microbubbles acted on PLGA/TCP 3D-printing scaffolds to promote cell adhesion and proliferation and to promote bone regeneration and repair. Type I collagen is secreted by osteoblasts and expressed in the formation and maturation of the extracellular matrix of osteoblasts, and it is an important indicator of early osteogenesis [28, 29]. ALP is an important regulatory substance involved in bone formation and bone metabolism, and it is positively correlated with the differentiation and maturation of osteoblasts. ALP is an extracellular enzyme representing osteoblasts [30]. In this experiment, the expression of type I collagen and ALP activity in the LIPUS combined with lipid microbubble group increased at 14 days, indicating that LIPUS promoted the expression of type I collagen and ALP activity at different stages, which is consistent with previous studies [31]. Scanning electron microscopy showed that when cultured for 21 days, the proliferation and osteogenesis of BMSCs in the LIPUS combined with lipid microbubbles group were significantly higher than those in the other groups, indicating that LIPUS combined with lipid microbubbles can promote the adhesion and proliferation of BMSCs in PLGA/TCP 3D printing scaffolds.

### Limitations

Irradiation of PLGA/TCP 3D-printed scaffolds with LIPUS combined with lipid microbubbles can enhance the proliferation and osteogenesis of BMSCs. The technique can be used as a potential adjuvant treatment tool for bone regeneration and provides a new idea for the application of bone tissue engineering as well as broad research prospects for clinical bone defect regeneration therapy.

## Data Availability

The datasets used and/or analysed during the current study available from the corresponding author on reasonable request.

## Abbreviations

LIPUS: low-intensity pulsed ultrasound
MBs: microbubbles
SI: sonic intensity
BMSCs: bone marrow mesenchymal stem cells
PLGA: poly lactic-glycolic acid copolymer
TCP: tricalcium phosphate
CDHA: calcium-deficient hydroxyapatite
UV: ultraviolet
ALP: Alkaline phosphatase
qPCR: quantitative polymerase chain reaction.
